# ASAMS: An Adaptive Sequential Sampling and Automatic Model Selection for Artificial Intelligence Surrogate Modeling

**DOI:** 10.3390/s20185332

**Published:** 2020-09-17

**Authors:** Carlos A. Duchanoy, Hiram Calvo, Marco A. Moreno-Armendáriz

**Affiliations:** 1 Instituto Politécnico Nacional, Centro de Investigación en Computación, Av. Juan de Dios Bátiz s/n, Ciudad de México 07738, Mexico; hcalvo@cic.ipn.mx (H.C.); mam_armendariz@cic.ipn.mx (M.A.M.-A.); 2 Cátedra CONACyT, Instituto Politécnico Nacional, Centro de Investigación en Computación, Av. Juan de Dios Bátiz s/n, Ciudad de México 07738, Mexico

**Keywords:** surrogate model, adaptive sequential sampling, machine learning

## Abstract

Surrogate Modeling (SM) is often used to reduce the computational burden of time-consuming system simulations. However, continuous advances in Artificial Intelligence (AI) and the spread of embedded sensors have led to the creation of Digital Twins (DT), Design Mining (DM), and Soft Sensors (SS). These methodologies represent a new challenge for the generation of surrogate models since they require the implementation of elaborated artificial intelligence algorithms and minimize the number of physical experiments measured. To reduce the assessment of a physical system, several existing adaptive sequential sampling methodologies have been developed; however, they are limited in most part to the Kriging models and Kriging-model-based Monte Carlo Simulation. In this paper, we integrate a distinct adaptive sampling methodology to an automated machine learning methodology (AutoML) to help in the process of model selection while minimizing the system evaluation and maximizing the system performance for surrogate models based on artificial intelligence algorithms. In each iteration, this framework uses a grid search algorithm to determine the best candidate models and perform a leave-one-out cross-validation to calculate the performance of each sampled point. A Voronoi diagram is applied to partition the sampling region into some local cells, and the Voronoi vertexes are considered as new candidate points. The performance of the sample points is used to estimate the accuracy of the model for a set of candidate points to select those that will improve more the model’s accuracy. Then, the number of candidate models is reduced. Finally, the performance of the framework is tested using two examples to demonstrate the applicability of the proposed method.

## 1. Introduction

Many science and engineering fields rely on computer simulations to replace expensive physical experimentation to analyze and improve the quality of different designs, methodologies, or products. The continuous research of numerical simulations has reduced the gap between the physical system and its model. Nevertheless, this improvement comes with a cost in time due to the complexity of such numerical models. Surrogate modeling has become a solution for approximating the expensive numerical simulations of complex systems used to solve heavily iterative problems, such as optimization problems, and achieve acceptable accuracy at a low computational cost.

Surrogate modeling has been incorporated in multiple fields. In [1], the authors develop a multi-fidelity surrogate model for a microwave component. In [2] the authors use a surrogate Kriging Model to represent bridge structures. In [3], surrogate models have been used for control design and feedback prediction. They have alsobeen used in pedestrian detection in [4] or for process analysis in industrial plants in [5].

Continuous research fueled artificial intelligence, developing new algorithms that, together with large amounts of information, are capable of imitating the behavior or decision-making of complex systems or processes.

These advances have also been caught in the potential of the surrogate models, allowing them to extend their use to different disciplines, such as Digital Twins (DT), Design Mining (DM) and Soft Sensing. DT are virtual replicas of physical systems generally developed to analyze and optimize such systems. While this technology shares some of the same principles of surrogate models, it is still at its early development stage [6]. The integration of big data and artificial intelligence models support DT success for their potential and intense impact in multiple fields. Along with their successful performance in some applications [7,8], DT can benefit from methodologies developed for surrogate modeling.

Design Mining (DM) uses Artificial Intelligence techniques to iteratively search the attribute space of a physical object evaluated directly through rapid prototyping, which is generally expensive, and commonly surrogate models are used to reduce the physical system sampling [9]. DM explores the design space evaluating directly through rapid prototyping in systems in which there are no formal models or the computational models are too expensive and imprecise. Nonetheless, this methodology comes with a considerable cost in time and resources. While DM considers rapid prototyping as a fundamental part of the exploration of the design space, surrogate modeling has been proposed as the main alternative to reduce the cost related to this methodology [10,11,12].

Soft sensors make predictions of observable variables whenever hardware sensors are unfeasible. They are surrogate models of the system that process several related signals of hardware sensors to estimate another variable’s value and have the advantage of a fast response at a low cost. Some of its applications are fault detection [13], real-time monitoring [14], complex motion capture [15], and sensor validation. Data-driven soft sensors perform well if the training data and the testing data have the same distribution, which is generally not accomplished in real-world industrial applications [16]. These methodologies have driven surrogate models to different horizons, but also they have generated new research opportunities. The artificial intelligence surrogate models need to handle complex model selection and hyperparameter tuning while keeping the sample data points to a minimum. This particular problem motivates us to develop an adaptative sampling methodology for artificial intelligence data-driven models.

The rest of this paper is organized as follows. In Section 2 we review what we consider the most relevant related work with our proposal and state the paper contribution. In Section 3 we explain in detail the architecture of our proposal, the initial static sampling, the design of the AutoML reduction method, and the adaptive sampling methodology. In Section 4 we present several test cases, including software integrations. In Section 5, we give details on the methodology’s performance. Finally, Section 6 is devoted to the concluding remarks.

## 2. Related Work

All surrogate modeling techniques share the same objective representing the target system as accurately as possible. This objective can be archived by increasing the size of the training data set to get a better understanding of the complete system, as models constructed using bigger datasets record better accuracy [17]. However, this approach can become very expensive due to the computational cost or economic cost of sampling the target plant. In these cases, a second objective is introduced, which is to minimize the cost associated with measuring the target system. These surrogate modeling cases are stated as multiobjective optimization problems, whose goals are to build a model as accurate as possible whit the minimum number of points as possible. The search for the best compromise has been studied from different angles. The first approach, *meta-modeling*, focuses on tailoring the model that will be used to emulate the target system. This is done through careful model selection and hyperparameter tuning. The performance of the most prominent methods for surrogate creation depends more strongly on the correct setting of many internal hyperparameters [18], and the correct selection of the modeling method is critical for achieving good performance [19]. The second approach, *sampling*, focuses on a sampling strategy that determines the best data points to measure the target phenomena. It has been proven that the choice of observed points is crucial to the prediction quality of the model.

The meta-modeling approach focuses on the strong sensitivity-to-design decisions during the construction of machine learning surrogates. The main problems are model selection and hyperparameter tuning. The field of AutoML aims to make these decisions in an automated way [20]. Every machine learning system has hyperparameters, and the most basic task of AutoML is to automatically optimize these parameters; this is referred as *automatic hyperparameter optimization* (HPO). The traditional way of performing HPO is through the grid search algorithm [21], which performs an exhaustive search across a grid of parameters comparing them via a distance metric. Even though this is an old algorithm, it is relatively simple and is still one of the most used nowadays [22,23,24].

The sampling approach is a crucial process in constructing an accurate surrogate model. In [25], the authors classified the main sampling approaches into two categories: one-shot sampling methods and adaptive sequential sampling. One-shot sampling methods consist of generating sampling points through different design of experiments (DoE) methods. Their objective is to allocate the sampling points reasonably as uniformly as possible in the design space. Classic DoE methods include Factorial Designs [26], Central Composite Design (CCD) [27], Monte Carlo Sampling (MCS) [28] and Latin Hypercube Design (LHD) [29]. Adaptive Sampling distributes more points in the regions of interest by analyzing the performance of the surrogate model in previous data. In comparison, adaptive sampling performs better than the one-shot sampling, having great potential to build accurate meta-models with fewer points [30]. For complex systems, sampling more points where the surrogate model has large prediction errors increases the accuracy using fewer points than those needed if the points are sampled evenly; that is, focusing in regions with large prediction errors (regions of interest) allowing the sampling process to adapt to the target function. For improving the surrogate model accuracy, the selection of the regions of interest must consider two conflicting parts: (1) local exploitation: using the model to find regions with large prediction error and (2) global exploration to discover interesting regions that have not been sampled before [31].

The current adaptive sampling approaches are classified into four categories based on the representation type of prediction errors. The *variance-based adaptive sampling* [31,32,33,34] uses estimations of statistical models, mainly the Kriging model, to detect the regions of interest of regression models. The *query-by-committee (QBC)* strategy [31,35,36] uses the predictions of multiple competing surrogate models as a committee to predict the response at a candidate point. The *cross validation (CV) based adaptive sampling* [37,38,39] estimates the prediction error at a candidate point using a cross-validation process. Finally, The *gradient-based adaptive sampling* [40,41,42] uses the local gradient information of the model to represent the prediction errors. All adaptive sampling methodologies use a particular local exploitation method for searching for new critical samples accordingly to the meta-model performance. The variance-based and gradient-based approaches use the model information as the model gradient or the model hyperparameters, which represent a model dependence. This limits the models that can be used for surrogating, mainly to the Kriging models, while query-by-committee, and cross-validation can be applied to different types of surrogate models.

The main limitation of the current adaptive sampling methods is that they seek to identify the points that most favor a predetermined meta-model based on its performance [37,38,39,40,41,42]. However, it is not easy to determine if the points were selected due to the target system behavior or to an intrinsic limitation of the selected meta-model. Ideally, all the selected samples are critical due to the complex behavior of the target system; however, when evaluated through their performance in the surrogate model, it is not possible to differentiate if they are complex by themselves or if they are only complex for the selected model. This issue raises the following question: does the selected meta-model and its current hyperparameter selection is suitable for representing the target system? or a different meta-model with a different tuning can improve the performance of the surrogate? The question of whether it is possible to generate a new sample point through an adaptive sampling methodology that is independent of the meta-model used to evaluate its performance.

To solve this problem, we propose an adaptive sampling methodology that combines the CV and QBC methodologies together with a grid search algorithm to generate new sampling points at the same time that performs a model selection and a hyperparameter tuning.

## 3. Proposed Method (ASAMS)

**Figure 1:**
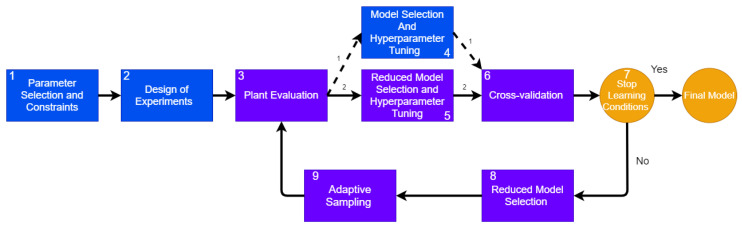
Adaptive Sampling and Automatic Model Selection (ASAMS) general architecture.

The proposed methodology is named *Adaptive Sampling and Automatic Model Selection* (ASAMS). It consists of an AutoML methodology to adjust the hyperparameters of the AI algorithms, and an adaptive sampling method that combines the two methods independently of models CV and QBC. The AutoML method is complemented with a reduction process based on the elitism mechanism of evolutionary methods. A flow diagram of the methodology consists of the modules shown in Figure 1, explained in detail below.

1. Parameter Selection and Constraints. In this stage, the problem statement is carried out, determining the design parameters and constraining the design space. See Section 3.2 for more details.
2. Design of experiments. In order to initialize the construction of the surrogate model, a small number of initial training points are generated using a one-shot sampling method or DoE. In this work, we decided to use a full factorial sampling but any other method can be applied.
3. Plant Evaluation. In this stage, the response of the plant to each training point is measured and assigned as a target for the surrogate training process. The process of evaluating the plant can be online as mathematical models, computational simulations, or physical online measurements, as in DT; or it can be offline for DM. In this paper, we analyze two problems that use an online implementation: one mathematical model and one multiphysics computational simulation.
4. Model Selection and Hyperparameter Tuning. In this step, an AutoML algorithm is applied to perform an algorithm selection and hyperparameter tuning for each possible algorithm. See Section 3.3 for more details.
5. Reduced Model Selection and Hyperparameter Tuning. This step is similar to the Model Selection and Hyperparameter Tuning with only one difference: after the first iteration, the number of candidate models will be reduced through an elitism mechanism; this step performs the hyperparameter tuning and model selection to reduce candidates.
6. Cross-validation. As a result, this process returns the best candidates and the validation score for each candidate model obtained by cross-validation.
7. Stop Learning Conditions. In this step, the methodology validates if the algorithm has met any stop criteria. In this proposal, we consider three different stop conditions. See Section 3.4 for more details.
8. Reduced Model Selection. The process of selecting a suitable model and the correct hyper-parametrization can be explained as an exploration–exploitation problem. During the Model Selection and Hyperparameter Tuning step, the design space is explored, and in the Reduced Model Selection phase, we propose an exploitation mechanism to reduce the search space. See Section 3.5 for a detailed explanation.
9. Adaptive Sampling. In this step, a novel mechanism of adaptive sampling that combines CV and QBC generates new training points through a Voronoi approach. See Section 3.7 for a detailed explanation of the contribution.

We developed the ASAMS algorithms mainly in Python [43], with the idea of integrating them with computer-aided engineering (CAE) and computer-aided design (CAD) software. We performed the integration through Matlab^®^ [44]. As CAE software, we selected COMSOL Multiphysics^®^ [45] and as CAD software we used SolidWorks^®^ [46]. Software and experiments are available at GitHub [47]. Some references are made to the code implementation, however, the methodology is independent of the software selection.

### 3.1. Formal Problem Statement

As mentioned in Section 2, the surrogate model creation problem can be stated as an optimization problem which consists of minimizing the error between meta-model prediction and the real measurement represented by Equation (1), and minimizing the number of experiments *M* Equation (2), subjected to the algebraic and inequality restriction in functions of the design parameters Equations (3) to (12).

(1) $$min(\frac{1}{N}\sum_{N}^{i=1}{\left(Y^{i}-{\bar{Y}}^{\left(i,t\right)}\right)}^{2})$$

(2) min(M)

(3) $$\begin{array}{cc} {\bar{Y}}^{\left(i,t\right)} & =F_{t}\left(Hp_{\left(v,t\right)},X_{i},T_{d}\right) \end{array}$$

(4) $$\begin{array}{ccc} \hat{FT} & =\{F_{1},F_{2},F_{3},\ldots ,F_{t}\} & t=1,2,3,\ldots ,T \end{array}$$

(5) $$\begin{array}{ccc} \hat{Hp} & =\{{\hat{Hp}}_{1},{\hat{Hp}}_{2},{\hat{Hp}}_{3},\ldots ,{\hat{Hp}}_{t}\} & \end{array}$$

(6) $$\begin{array}{ccc} {\hat{Hp}}_{t} & =\{Hp_{\left(1,t\right)},Hp_{\left(2,t\right)},Hp_{\left(3,t\right)},\ldots ,Hp_{\left(v_{t},t\right)}\} & v_{t}=1,2,3,\ldots ,V_{t} \end{array}$$

(7) $$\begin{array}{ccc} Hp_{\left(v,t\right)} & =\{hp_{\left(1,t\right)},hp_{\left(2,t\right)},hp_{\left(3,t\right)},\ldots ,hp_{\left(k_{t},t\right)}\} & k_{t}=1,2,3,\ldots ,K_{t} \end{array}$$

(8) $$\begin{array}{ccc} X_{i} & =\{x_{\left(1,i\right)},x_{\left(2,i\right)},x_{\left(3,i\right)},\ldots ,x_{\left(j,i\right)}\} & i=1,2,3,\ldots ,N \end{array}$$

(9) $$\begin{array}{ccc} Y_{i} & =\{y_{\left(1,i\right)},y_{\left(2,i\right)},y_{\left(3,i\right)},\ldots ,y_{\left(s,i\right)}\} & s=1,2,3,\ldots ,S \end{array}$$

(10) $$\begin{array}{ccc} T_{d} & =\{d_{1},d_{2},d_{3},\ldots ,d_{m}\} & m=1,2,3,\ldots ,M \end{array}$$

(11) $$\begin{array}{ccc} hp_{\left(k_{t},t\right)} & \in {\hat{P}}_{\left(k_{t},t\right)}\;\;{\hat{P}}_{\left(k_{t},t\right)}=\left\{p_{\left(1,k_{t},t\right)},p_{\left(2,k_{t},t\right)},p_{\left(3,k_{t},t\right)},\ldots ,p_{\left(q_{\left(k,t\right)},k_{t},t\right)}\right\} & q_{\left(k,t\right)}=1,2,3,\ldots ,Q_{\left(k,t\right)} \end{array}$$

(12) $$\begin{array}{ccc} x_{j}^{L} & \leq x_{j}\leq x_{i}^{U} & j=1,2,3,\ldots ,J \end{array}$$

The target error, Equation (1), is calculated using the outputs of the surrogate model ${\bar{Y}}^{\left(i,t\right)}$ and the corresponding targets of the testing dataset $Y^{i}$ of *N* elements, in which every element is a set of *S* outputs, as shown in Equation (9). The *i*-th output of the surrogate model ${\bar{Y}}^{\left(i,t\right)}$ is a function of the selected model $F_{t}$ performance, Equation (3), which depends on the training data set $T_{d}$ used to fit the model; the hyperparameters $Hp_{\left(v,t\right)}$ selected for the model and the *i*-th input vector $X_{i}$ of the testing dataset, Equation (8). The other target is the minimization of the number of points *M* of the training dataset $T_{d}$, shown in Equation (10).

The design variables selected for this optimization problem are the surrogate model algorithm $F_{t}$ from a set of possible *T* models $\hat{FT}$, stated in Equation (4). The hyperparameters set $Hp_{\left(v,t\right)}$, shown in Equation (7), are used to configure the $F_{t}$ model. Each hyperparameter set can have $K_{t}$ different hyperparameters, and each $hp_{\left(k,t\right)}$ hyperparameter is selected from a set $P_{\left(k_{t},t\right)}$ of possible hyperparameter values, Equation (11).

Finally, we must consider that all the model inputs are constrained, as represented by Equation (12). The nomenclature used in this section is shown in the end of this paper.

### 3.2. Parameter Selection and Constraints

A correct selection of the design parameters and a constrained search space are crucial factors for developing an accurate surrogate model. In this step, we define the initial size *M* and data points of the initial dataset $T_{d}$, as well as the size *N* and input $X_{i}$ and output $Y^{i}$ vectors of the testing dataset. Finally, we define the number *J*, type, and constraints for each input parameter using a JSON notation built by a list of objects representing each design parameter. In this proposal, we consider three different types of input variables: continuous, discrete, and categorical. Continuous and discrete variables are constrained by a minimum and a maximum value, while categorical ones are constrained to a set of values. Categorical variables are defined by two properties: set and table. Set is described by a list of categories and table has a list of objects associated with their corresponding list of values. Listing 1 shows an example of these parameters.

### 3.3. Model Selection and Hyperparameter Tuning

The main inconvenience of the grid search is its high dimensionality and time cost. Still, its required execution time is relatively short compared with complex computational simulations of physical experimentation. This motivated us to incorporate an exhaustive grid search for hyperparameter tuning for multiple algorithms—this allows the algorithm to perform hyperparameter tuning and model selection simultaneously. With these, we seek to give the surrogate model the greatest possibility to obtain a suitable performance with the minimum number of plant evaluations. In our implementation, we selected a set $\hat{FT}$ of three machine learning algorithms: a support vector machine regressor (SVM)  [48], a random forest regressor (RF)  [49], and a Bayesian Ridge model (BR) [50], as shown in Equation (13), although this methodology can be generalized to any set of machine learning algorithms.

We also make a proposal for the hyperparameters for each algorithm in Listing 2. The formal statement of the hyperparameters is shown in Equations (14) to (28).

(13) $$\begin{array}{ccc} \hat{FT} & =\{SVM,RF,BR\}\; & \end{array}$$

(14) $$\begin{array}{ccc} Hp_{SVM} & =\{kernel,C,gamma\}\; & \end{array}$$

(15) $$\begin{array}{ccc} Hp_{RF} & =\left\{n\_estimators,max\_features,max\_depth,min\_samp\_leaf,min\_samp\_split\right\}\; & \end{array}$$

(16) $$\begin{array}{cc} Hp_{BR} & =\{alpha\_1,alpha\_2,lambda\_1,lambda\_2\} \end{array}$$

(17) $$\begin{array}{cc} kernel & \in \{‘linear^{’},‘rbf^{’}\} \end{array}$$

(18) $$\begin{array}{cc} C & \in \{0.01,0.1,1,10,100\} \end{array}$$

(19) $$\begin{array}{cc} gamma & \in \{1,10,100,1000,10000{,}^{\prime }auto^{\prime }\} \end{array}$$

(20) $$\begin{array}{cc} n\_estimators & \in \{200,600,1000,1400,1800\} \end{array}$$

(21) $$\begin{array}{cc} max\_features & \in \{‘auto^{’},‘log2^{’}\} \end{array}$$

(22) $$\begin{array}{cc} max\_depth & \in \{10,50,90,None\} \end{array}$$

(23) $$\begin{array}{cc} min\_samples\_leaf & \in \{1,2,4\} \end{array}$$

(24) $$\begin{array}{cc} min\_samples\_split & \in \{2,5,10\} \end{array}$$

(25) $$\begin{array}{cc} alpha\_1 & \in \{1\times 10^{-4},1\times 10^{-6},1\times 10^{-7}\} \end{array}$$

(26) $$\begin{array}{cc} alpha\_2 & \in \{1\times 10^{-4},1\times 10^{-6},1\times 10^{-7}\} \end{array}$$

(27) $$\begin{array}{cc} lambda\_1 & \in \{1\times 10^{-4},1\times 10^{-6},1\times 10^{-7}\} \end{array}$$

(28) $$\begin{array}{cc} lambda\_2 & \in \{1\times 10^{-4},1\times 10^{-6},1\times 10^{-7}\} \end{array}$$

In each iteration, the grid search algorithm will test the performance of all possible hyperparameter combinations for each candidate model. In the proposed structure of models and hyperparameters, this will represent 60 hyperparameter combinations for the SVM algorithm, 360 hyperparameter combinations for the RF algorithm, and 81 hyperparameter combinations for the BR algorithm.

The combinations of all the algorithms are represented in $\hat{Hp}$ show in Equations (29)–(32).

(29) $$\begin{array}{ccc} \hat{Hp} & =\{{\hat{HPC}}_{SVM},{\hat{HPC}}_{RF},{\hat{HPC}}_{BR}\} & \end{array}$$

(30) $$\begin{array}{ccc} {\hat{HPC}}_{SVM} & =\{HP_{SVM}^{1},HP_{SVM}^{2},HP_{SVM}^{3},\ldots ,HP_{SVM}^{E_{1}}\} & E_{1}=60 \end{array}$$

(31) $$\begin{array}{ccc} {\hat{HPC}}_{RF} & =\{HP_{RF}^{1},HP_{RF}^{2},HP_{RF}^{3},\ldots ,HP_{RF}^{E_{2}}\} & E_{2}=360 \end{array}$$

(32) $$\begin{array}{ccc} {\hat{HPC}}_{BR} & =\{HP_{BR}^{1},HP_{BR}^{2},HP_{BR}^{3},\ldots ,HP_{BR}^{E_{3}}\} & E_{3}=81 \end{array}$$

### 3.4. Stop Conditions of Learning

We decided to stop the surrogate optimization process by three different criteria. The first criterion is the accuracy assessment (acc). It considers if the surrogate has achieved the desired performance by performing an error comparison using Equation (33).

(33) $$acc=\left\{\begin{array}{c} True:min\left(\vec{\varepsilon_{m}}\right)\leq \varepsilon_{t}\\ False:min\left(\vec{\varepsilon_{m}}\right)>\varepsilon_{t} \end{array}\right.$$

where $\vec{\varepsilon_{m}}=\left[{\varepsilon_{m}}_{1},{\varepsilon_{m}}_{2},\ldots ,{\varepsilon_{m}}_{i}\right]$ is the vector of the error ${\varepsilon_{m}}_{i}$ for each of the *i* models; $\varepsilon_{t}$ is the target error.

The second criterion limits the computational power expended by the search algorithms constraining the maximum number of iterations ($max_{I}$). The third criterion considers the cost in time or money in each plant evaluation, limiting the maximum number of points ($max_{P}$) that can be evaluated.

### 3.5. Reduced Model Selection

The proposed sequential methodology changes the number of training points in each iteration, which gives more information to the machine learning models, enabling us to change the model that fits better for solving the target problem. However, the grid search algorithm suffers from the dimensionality problem and it is necessary to reduce the models that are too far away to represent the intended phenomenon. This characteristic can be interpreted as an exploration–exploitation problem in which we want to keep exploring the models that have an opportunity to fit the system but focus on exploiting the ones that have better performance. Previously, the exploration process through Model Selection and Hyperparameter Tuning was discussed in Section 3.3 in which the proposed structure of models and hyperparameters represent a total of 501 models. However, testing all the models in each iteration of the ASAMS is too computationally expensive. This motivated us to include a mechanism for reducing the number of candidate models in each iteration.

We developed an exploitation mechanism based on elitism. The main objective is to reduce the models to explore via grid search in each iteration. The proposed exploitation mechanism is Reduced Model Selection. As a first step, we rank the models in $\hat{FT}$ for all ${\hat{HP}}_{t}$ hyperparameter combinations taking advantage of the CV score obtained by the grid search method. Then, we proceed to remove the models that had the worst performance. The number of models to be removed in each iteration is provided as a hyperparameter of ASAMS. Then, after each hyperparameter set ${\hat{HP}}_{t}$ is sorted, we proceed to remove the $W_{t}$ hyperparameter combinations from each model $F_{t}$ that had the worst performance. The number of models $W_{t}$ to be removed in each iteration is a function of the keepRate hyperparameter of ASAMS, shown in Equation (34).

(34) $$W_{t}=round\left(\left(1-keepRate\right)V_{t}\right)$$

As an example, we propose keepRate=0.6, which means that we will keep 60% of the hyperparameter combinations, using the proposed method described in Section 3.3. We estimate the $W_{t}$ values for three subsequent iterations in Equations (35) to (43).

In the first iteration, we assume that we have the 501 initial models, and reduce them considering the proposed keep ratio, as indicated in Equations (35)–(37).

(35) $$\begin{array}{cc} W_{SVM} & =round\left(\left(1-keepRate\right)\cdot V_{SVM}\right)=round\left(\left(1-0.6\right)\cdot 60\right)=24 \end{array}$$

(36) $$\begin{array}{cc} W_{RF} & =round\left(\left(1-keepRate\right)\cdot V_{RF}\right)=round\left(\left(1-0.6\right)\cdot 360\right)=144 \end{array}$$

(37) $$\begin{array}{cc} W_{BR} & =round\left(\left(1-keepRate\right)\cdot V_{BR}\right)=round\left(\left(1-0.6\right)\cdot 81\right)=32 \end{array}$$

In the second iteration, we need to remove the models selected in the first iteration, which leaves us 36 SVM, 216 RF, and 49 BR models. Then we will reduce them further in the second iteration considering the proposed keep ratio, as indicated in Equations (38)–(40).

(38) $$\begin{array}{cc} W_{SVM} & =round\left(\left(1-keepRate\right)\cdot V_{SVM}\right)=round\left(\left(1-0.6\right)\cdot 36\right)=14 \end{array}$$

(39) $$\begin{array}{cc} W_{RF} & =round\left(\left(1-keepRate\right)\cdot V_{RF}\right)=round\left(\left(1-0.6\right)\cdot 216\right)=58 \end{array}$$

(40) $$\begin{array}{cc} W_{BR} & =round\left(\left(1-keepRate\right)\cdot V_{BR}\right)=round\left(\left(1-0.6\right)\cdot 49\right)=20. \end{array}$$

In the third iteration, we need to remove the models selected in the first and second iteration, which leaves us 22 SVM, 158 RF, and 29 BR models. Then, we will reduce them further in the third iteration considering the proposed keep ratio, as indicated in Equations (41)–(43).

(41) $$\begin{array}{cc} W_{SVM} & =round\left(\left(1-keepRate\right)\cdot V_{SVM}\right)=round\left(\left(1-0.6\right)\cdot 22\right)=9 \end{array}$$

(42) $$\begin{array}{cc} W_{RF} & =round\left(\left(1-keepRate\right)\cdot V_{RF}\right)=round\left(\left(1-0.6\right)\cdot 158\right)=63 \end{array}$$

(43) $$\begin{array}{cc} W_{BR} & =round\left(\left(1-keepRate\right)\cdot V_{BR}\right)=round\left(\left(1-0.6\right)\cdot 29\right)=12 \end{array}$$

This process is repeated for all iterations.

### 3.6. Adaptive Sampling

The main challenge is to build a suitable surrogate model with the minimum number of training examples, which means we cannot waste samples or system evaluations. It is particularly meaningful if assessments come from a physical system that is constructed and measured. We propose a framework that partitions the sampling space into regions in Section 3.6.1, and then decides which the best are, via a CV and QBC evaluation (Section 3.6.2), and take few candidate points from them.

#### 3.6.1. Partition of the Sampling Space and Candidate Points Selection

We define the sampling region as the set of input values that are valid in all the input constraints. At the start, the sampling region must be reduced from an infinite number to a finite number of points. Therefore, the selected sampling points named candidate points should be far enough from each other and spread in all the search space. In [51], the authors generate a candidate points population using Monte Carlo sampling (MCS); other alternatives are translational propagation Latin Hypercube Design (TPLHD) [52], Uniform Design (UD) [53], and Voronoi sampling [39]. From the latest emerging methodologies, we selected the Voronoi sampling approach because the partition of the design space is done according to the current samples, which allows us to focus on the regions of interest instead of the whole design space.

In our proposal, we create Voronoi regions based on the work of [39] from the training samples and incorporate a constraint-handling technique to select a subset of Voronoi vertexes that meet the parameters’ constraints. For constraint handling, the methodology considers two approaches: for any Voronoi vertex that goes outside the minimum and maximum value of any parameter, we apply the death penalty constraint-handling technique [54], in any other point we apply the bounce back constraint-handling technique [55]. The set of data points selected by this technique is denominated as the Voronoi set (VoP) and is represented in Equation (44).

(44) $$VoP=\{vp_{1},vp_{2},vp_{3},\ldots vp_{g}\}\;\;\;g=1,2,3,\ldots G$$

**Figure 2:**
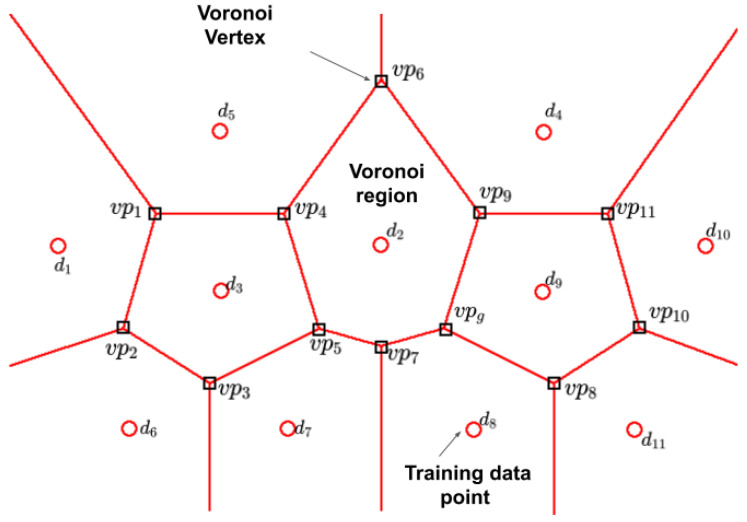
Voronoi plane diagram [56].

The points of the Voronoi set are the Voronoi vertexes that are estimated from the points of the training dataset $T_{d}$. A graphical example of two-dimensional Voronoi regions is shown in Figure 2.

#### 3.6.2. Region Assessment and Candidate Selection

The assessment process consists of estimating the precision of the surrogate model in each of the points of the Voronoi set VoP. In this proposal, we combine the two adaptive sampling approaches that have no model dependencies: the CV and the QBC approaches. Voronoi regions can be evaluated using the cross-validation approach proposed in [39]. However, we have more than one model trained with the same data as a result of the grid search methodology, and each model has a different assessment of regions of interest due to the particular characteristics of each model. For this reason, instead of using a particular model for estimating the critical regions, we decide to make a committee conformed from the best of each model type to determine the accuracy of each region. With this approach, we can use the leave-one-out cross-validation approach for each training point on each of the different models to estimate the global performance of all Voronoi regions.

In the first step, every Voronoi region *a* is rated considering the LOOCV score for the central point of the region by the best hyperparameter set $Hp_{B}$ for each model $F_{t}$ independently. As a result, every Voronoi region has as many ratings as different models trained by the grid search, as shown by Equations (45)–(46).

(45) $$\begin{array}{cc} {\bar{Y}}^{\left(a,t\right)} & =F_{t}\left(Hp_{B},X_{a},T_{d}\cap \left\{X_{a}\right\}\right) \end{array}$$

(46) $$\begin{array}{ccc} RR_{\left(a,t\right)} & ={\left(Y^{a}-{\bar{Y}}^{\left(a,t\right)}\right)}^{2} & t=1,2,3,\ldots ,T \end{array}$$

(47) $$\begin{array}{cc} {\hat{RR}}_{\left(a\right)} & =\frac{1}{T}\sum_{T}^{t=1}\left(RR_{\left(a,t\right)}\right) \end{array}$$

(48) $$\begin{array}{ccc} \hat{RR} & =\{{\hat{RR}}_{\left(1\right)},{\hat{RR}}_{\left(2\right)},{\hat{RR}}_{\left(3\right)},\ldots ,{\hat{RR}}_{\left(a\right)}\} & a=1,2,3,\ldots ,A \end{array}$$

where
$X_{a}$central point of the *a*-th Voronoi region$Hp_{B}$best hyperparameter set for the *t*-th model$T_{d}\cap \left\{X_{a}\right\}$Training data set excluding the $X_{a}$ point$RR_{\left(a,t\right)}$prediction error of the *t*-th model in the central point of the *a*-th region${\hat{RR}}_{\left(a\right)}$mean prediction error of the central point of the *a*-th region$\hat{RR}$set of mean prediction error of the all Voronoi regions${\bar{Y}}^{\left(a,t\right)}$Output vector of the *t*-th surrogate model with the *a*-th input vector trained excluding the $X_{a}$ pointAnumber of Voronoi regions


In order to consider the information of each model in the committee, the final score for each region is the mean value of the scores, see Equation (47).

**Figure 3:**
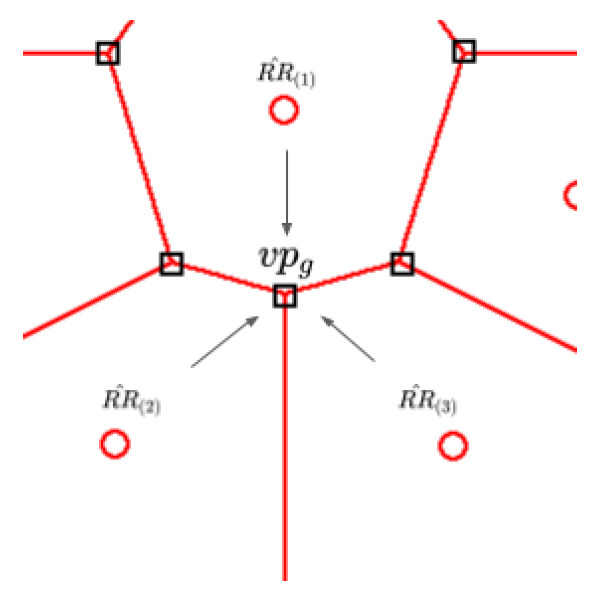
Vertex and colliding regions.

Finally, it is necessary to rate each candidate’s point. As we stated before, the candidate points are a subset of the Voronoi vertexes VoP. By definition, a Voronoi vertex is the midpoint where multiple Voronoi regions collide [56], as shown in Figure 3. The subset of regions that collide with the *g*-th Voronoi vertex ${\hat{RRz}}_{g}$ is shown in Equation (50). With this consideration, we define the performance of a candidate point $VPR_{g}$ as the mean performance of the adjacent regions (Equation (49)). After assessing the candidate points($\hat{VPR}$), we sort them and select the $N_{p}$ points with the worst performance. $N_{p}$ is a hyperparameter of ASAMS.

(49) $$\begin{array}{cc} VPR_{g} & =\frac{1}{Z}\sum_{Z}^{z=1}\left({\hat{RR}}_{\left(g,z\right)}\right) \end{array}$$

(50) $$\begin{array}{ccc} {\hat{RRz}}_{g} & =\{{\hat{RR}}_{\left(g,1\right)},{\hat{RR}}_{(g,2},{\hat{RR}}_{\left(g,3\right)},\ldots ,{\hat{RR}}_{\left(g,z\right)}\} & z_{g}=1,2,3,\ldots ,Z_{g} \end{array}$$

(51) $$\begin{array}{ccc} {\hat{RRz}}_{g} & \subseteq \hat{RR} & \end{array}$$

(52) $$\begin{array}{ccc} \hat{VPR} & =\{VPR_{1},VPR_{2},VPR_{3},\ldots ,VPR_{g}\} & g=1,2,3,\ldots ,G \end{array}$$

where
$VPR_{g}$assessment of the *g*-th Voronoi point${\hat{RRz}}_{g}$set of assessments of coliding regions to the *g*-th candidate Voronoi point$\hat{VPR}$set of assessments candidate Voronoi pointsGNumber of candidate Voronoi points$Z_{g}$Number of coliding regions to the *g*-th candidate Voronoi point


### 3.7. Step by Step Algorithm

In this section, we present a step-by-step algorithm to detail the input outputs and requirements of each step in the ASAMS Algorithm (see Section 3.7). The ASAMS algorithm uses the Problem or plant evaluation function, the parameters declaration, the candidate models Mdls, and the DoE algorithm as an input. It has five hyperparameters for adjusting the behavior of the algorithm. It uses a keep rate keepRate to indicate how many models must be carried on to the next iteration, the number of new points that will be generated each iteration nExp, and three stop conditions. The maximum number of points maxExp, the maximum number of iterations maxIter, and the target MSE Error.

The experiment design function creates the starting sample using a DoE and the parameter description. The plant evaluation function takes the sample and evaluates the problem. Then, the model selection and hyperparameter tuning (MdlSeletHyParm) function perform the grid search and the cross-validation of the candidate models. The stop condition function validates all the stop conditions and returns a boolean True if any condition has been broken; False in other cases. The reduce model function returns the remaining models after applying the keep rate. The adaptive sampling returns the nExp new points generated, and the joint function unites the new points and evaluations of the training set.   
**Algorithm 1** ASAMSdef ASAMS(Problem,Parameters,Mdls,DoE,
          keepRate,maxExp,maxIter,Error,nExp):
    Sample=ExperimentDesign(DoE,Parameters)
    SEval=PlantEvaluation(Problem,Sample)
    (mdlGrid MdlCVS)=MdlSeletHyParm(Sample,SSEval,Mdl)
    StopFlag=stopCondition(MdlCVS,maxExp,maxIter,Error)
    while StopFlag=True:
       (mdlGrid,MdlCVS)=ReducedModel(mdlGrid,MdlCVS,keepRate)
       newPoints=AdaptativeSampling(mdlGrid,Sample,SSEval,nExp)
       NPEval=PlantEvaluation(Problem,newPoints)
       (Sample,SSEval)=joint(Sample,SSEval,newPoints,NPEval)
       (mdlGrid MdlCVS)=MdlSeletHyParm(Sample,SSEval,mdlGrid)
       StopFlag=stopCondition(MdlCVS,maxExp,maxIter,Error)
    return mdlGrid

## 4. Case Studies 

### 4.1. Highly Nonlinear Oscillator

**Figure 4:**
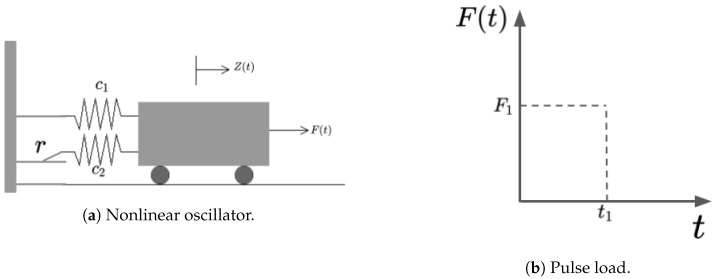
Nonlinear oscillator.

The first example is a highly nonlinear oscillator proposed in [57], as shown in Figure 4a, subjected to a rectangular load pulse with random duration and amplitude (Figure 4b). This is a benchmark problem widely used in many adaptive sampling works [34,58,59]. The limit state is defined by Equation (53), in which $z_{max}$ is the maximum displacement of the system and *r* is the displacement at which one of the spring yields. The maximum displacement $z_{max}$, Equation (54), is determined by the magnitude of the force $F_{1}$, the duration of the pulse $t_{1}$, the mass of the system *m*, and the oscillation frequency $w_{0}$. The oscillation frequency $w_{0}$ is defined by the spring constants $c_{1}$ and $c_{2}$. Performance function is defined by Equations (53)–(55) in Equation (56).

(53) $$g=3r-|z_{max}|$$

(54) $$z_{max}=\frac{2F_{1}}{mw_{0}^{2}}sin\left(\frac{w_{0}t_{1}}{2}\right)$$

(55) $$w_{o}=\sqrt{\frac{c_{1}+c_{2}}{m}}$$

(56) $$g\left(c_{1},c_{2},m,r,t_{1},F_{1}\right)=3r-|\frac{2F_{1}}{mw_{0}^{2}}sin\left(\frac{w_{0}t_{1}}{2}\right)|$$

**Figure 5:**
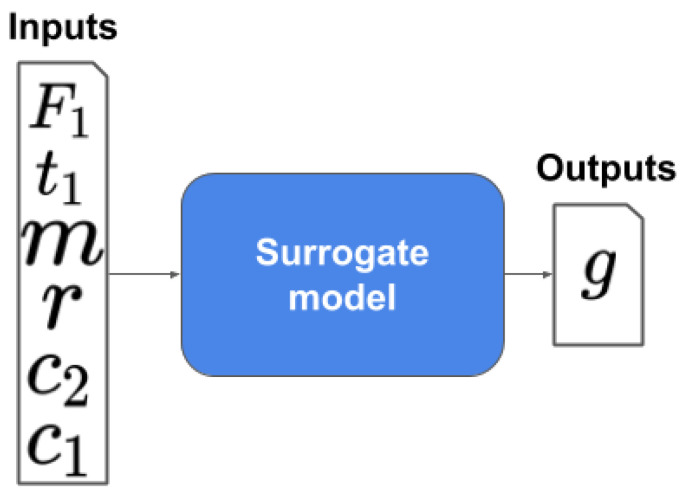
A nonlinear oscillator surrogate.

The surrogation problem stated to find a model $m_{ai}$ that is capable of representing the nonlinear oscillator performance (Equation (56)). The inputs and outputs of the proposed surrogate are presented in Figure 5 and Equation (57).

(57) $$m_{ai}\left(c_{1},c_{2},m,r,t_{1},F_{1}\right)=g$$

Parameter selection and constrains: this problem consists of five input variables whose constraints are defined in Listing 3 and is considered a moderate dimensional problem.

Design of experiments: we select the full factorial method for two levels with a total of 64 samples as starting training points.

Plant Evaluation: the plant evaluation will be performed using Equation (56).

Model and Hyperparameter constraints: the search of the surrogate model will be constrained to the algorithms and parameters proposed in Listing 2.

Selection of the stop Learning Conditions: the objective of this study is to compare the performance of the proposed algorithm with a baseline static design of experiments. As a baseline, we select a full factorial design of three levels which yields a total of 730 experiments. These motivate us to use as stop criteria a maximum number of points equal to the number of points of the factorial design 730. We select a maximum number of iterations of 100 and a target error of 0.

ASAMS parametrization: we select the following parameters for the ASAMS algorithm in this case study. A keep rate of 0.3, which represents that only 30% of the target models will be kept from each iteration. A number of experiments nExp of 8, which means that the algorithm will generate eight new experiments in or each iteration.

### 4.2. Magnetic Circuit

**Figure 6:**
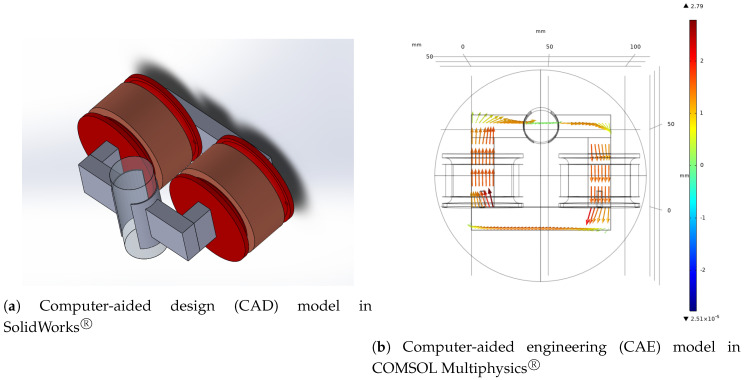
Magnetic circuit.

A magnetic circuit is a path in which a magnetic field can be enclosed, as shown in Figure 6a. These circuits can be modeled by computer simulation and have been used to optimize the circuit’s performance [60]. The magnetic circuit is described by a CAE simulation in COMSOL Multiphysics^®^, shown in Figure 6b. In this figure, the magnetic field of all the circuit is estimated using the CAE model. As the surrogate target, the mean magnetic field in the central tube is used.

**Figure 7:**
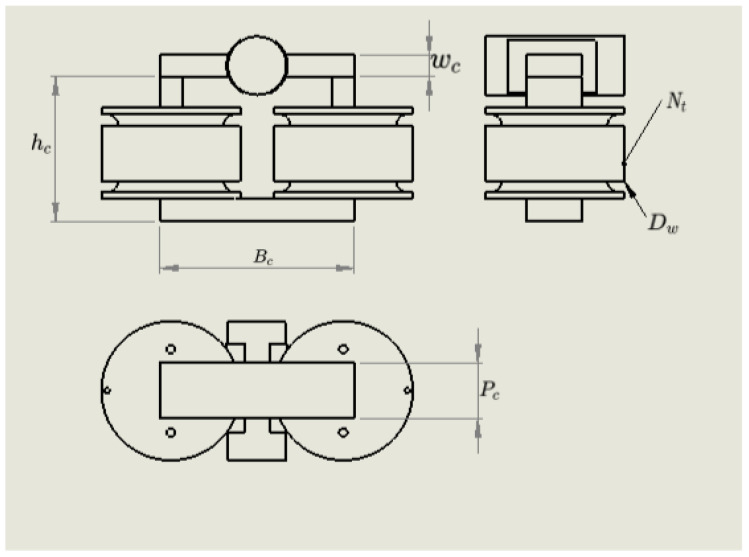
Geometrical parameters.

The physical parameters that determine the characteristics of the magnetic circuit are the number turns of the coil $N_{t}$, the core wire diameter $D_{w}$, and core geometry, defined by the core base $B_{c}$, the core height $h_{c}$, the core width $w_{c}$, and the core depth $P_{c}$. This problem consists of five input variables defined in Listing 3 and is considered a moderately-dimensional problem. Additionally to the geometrical parameters, it is necessary to include the electric current *I* that passes through the coils. Figure 7 shows the geometric parameters.

To state the model inputs, we take some considerations about the physical construction of the magnetic circuit, the core geometry is built using laminated silicon steel, which is commercially available in some specific geometries. For this reason, as a design parameters, we select a categorical parameter, the core Id that is related to the core geometry ($B_{c}$,$h_{c}$,$w_{c}$). The core wire diameter $D_{w}$ is limited to commercial gauges available and is considered a categorical parameter. The number of turns of the coil $N_{t}$ is considered a discrete integer parameter, and the core deep is a discrete parameter with increments of 0.5.

**Figure 8:**
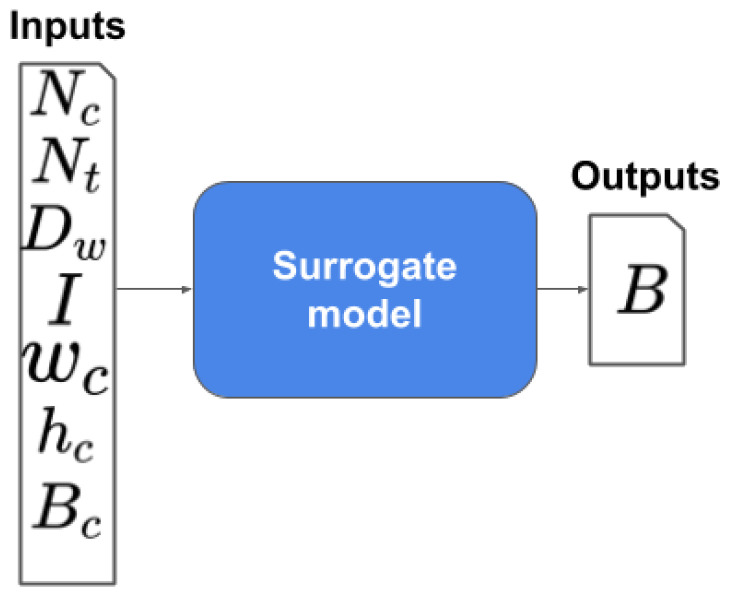
Magnetic circuit surrogate.

The objective is to find a surrogate capable of predicting the mean magnetic field *B* inside the tube at the center of the magnetic circuit. The proposed surrogate model is shown in Figure 8.

Parameter Selection and Constrains: the resulting five input variables and their constraints are defined in Listing 4.

Design of experiments: we select the full factorial method for two levels with a total of 33 samples as starting training points.

Plant Evaluation: the plant evaluation will be performed using the CAE simulation in COMSOL Multiphysics^®^.

Model and Hyperparameter constraints: the search of the surrogate model will be constrained to the algorithms and parameters proposed in Listing 2.

Selection of the stop Learning Conditions: the objective of this study is to compare the performance of the proposed algorithm with a baseline static design of experiments. As a baseline, we select a full factorial design of three levels which yields a total of 244 experiments. These motivate us to use, as stop criteria, a maximum number of points equal to the number of points of the factorial design 300. We selected a maximum number of iterations of 34 and a target error of 0.

ASAMS parametrization: we selected the following parameters for the ASAMS algorithm in this case study. A keep rate of 0.5, which represents that only 50% of the target models will be kept from each iteration. A number of experiments nExp of 14, which means that the algorithm will generate 14 new experiments in or each iteration.

## 5. Experiments and Discussion

### 5.1. Highly Nonlinear Oscillator

**Figure 9:**
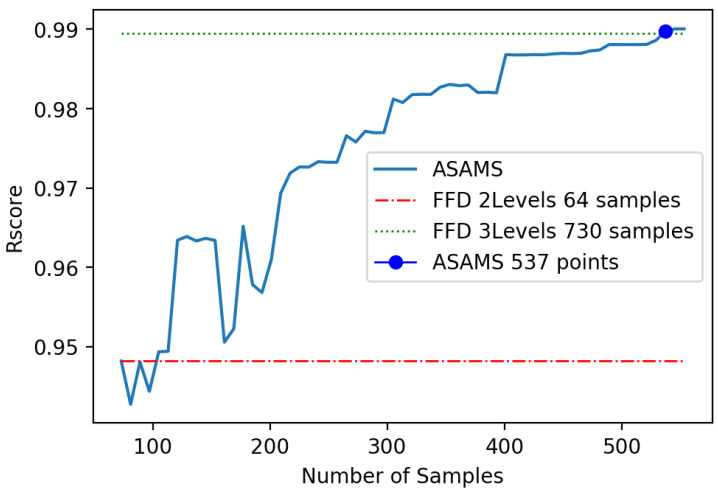
Baseline comparison of nonlinear oscillator.

The first problem is a benchmark problem; due to this reason, the performance of the algorithm can be analyzed by different means. First, we aim to compare the generalization capability of the algorithm trained using ASAMS with a one-shot approach as a baseline. We select the full factorial method for two and three levels that yields a total of 64 and 730 sampling points respectively. For validating the model performance, we will use a data set of 200 random points that are different from the ones used in the training of any algorithm. This testing set was used for comparing the performance of both baselines with the accuracy of the ASAMS at a different number of sample points. We select two different metrics for this comparison: the RScore metric [61] and the squared mean error (MSE) stated in Equation (58). In Figure 9, the comparison of the baselines with the ASAMS algorithm is shown. It can be appreciated that with 537 samples, the algorithm performs better than the 730 one-shot sampling approach.

**Figure 10:**
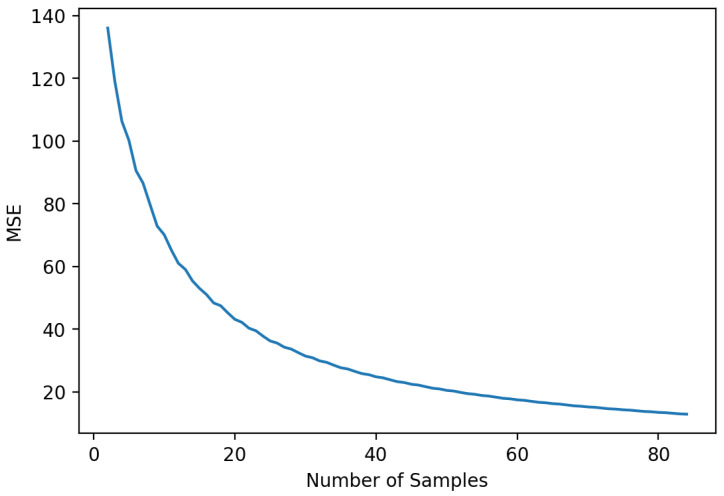
Cross-validation training score of nonlinear oscillator.

We noticed some fluctuations in the performance during the testing face between 150 and 250 samples; however, these variations were not present during the training cross-validation mean square error (MSE) shown in Figure 10.

**Figure 11:**
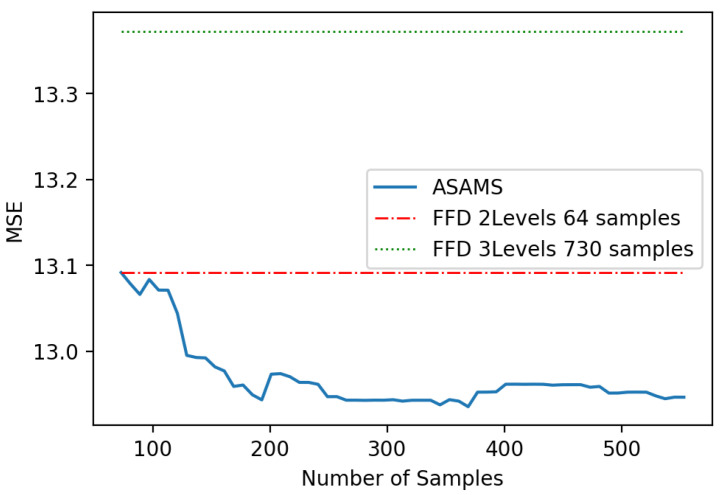
Interest region squared mean error (MSE) comparison of a nonlinear oscillator.

We found that the variability was due to the performance of the model in some particular zones. For this reason, we decided to focus our test on the zones of interest by generating additional testing points using the ASAMS methodology without training the model with them. The new testing set of fifty points was used to compare the models of ASAMS methodology for different numbers of samples with the baseline models. Results are shown in Figure 11. In this figure, we can appreciate that the type of model for each iteration is the same, which means that the ASAMS algorithm does not take advantage of the capability of changing the model while generating new training points.

The critical region experiment shows that the ASAMS model has better performance than any baseline in the regions that are considered as harder regions for the model to capture. Another important observation is that the baseline model with more points has the worst performance in the critical regions.

**Figure 12:**
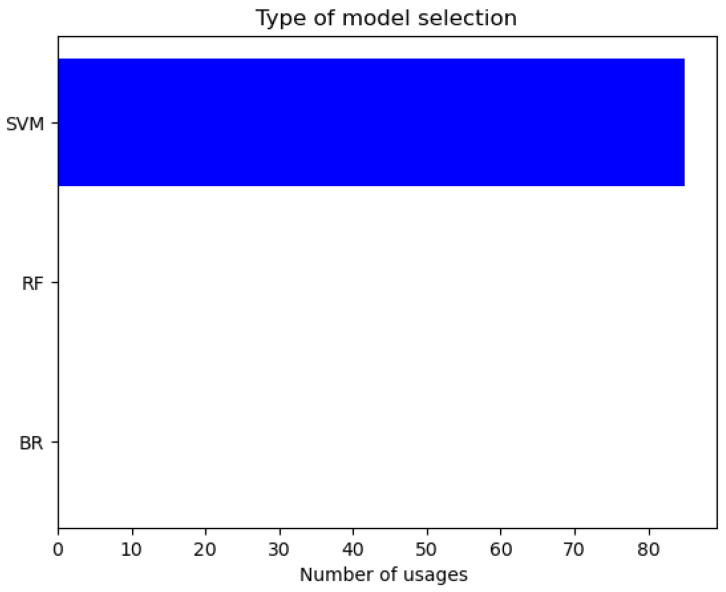
Type of model selection for the nonlinear oscillator.

One advantage of the ASAMS methodology is that it is capable of changing the type and hyperparameters of the selected surrogate model in each iteration. We hypothesize that this feature will help to improve the performance and the selected model will be changing when the size of the training dataset increases. To analyze this hypothesis, we decide to register the number of changes between the models. In Figure 12 we show the changes between the model types.

**Figure 13:**
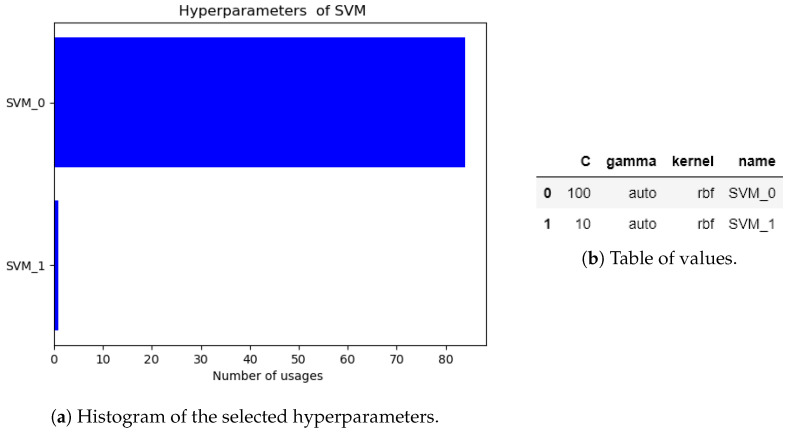
Histogram of the selected hyperparameters of the nonlinear oscillator.

It is clear that the algorithm has been fixed since the starting dataset. However, we also want to analyze if the algorithm at least changes the hyperparameters for fine-tuning the model. In Figure 13a, a histogram of the selected hyperparameters is shown. A table of the selected hyperparameters is shown in Figure 13b. In this analysis, we can appreciate that the algorithm adjusts a couple of times the hyperparameters before it converges to a specific set. The final model is SVM with the following hyperparameters: C = 100, gamma = auto, and kernel = rbf.

### 5.2. Magnetic Circuit

**Figure 14:**
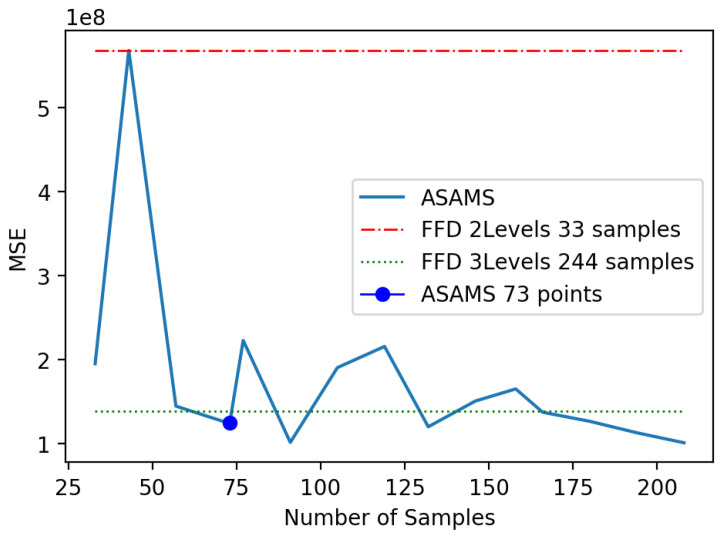
Baseline comparison of magnetic circuit.

This second problem is harder to evaluate because of the integration with COMSOL Multiphysics^®^ and SolidWorks^®^. In Figure 14 we compare the generalization capability of the algorithm trained using ASAMS with a one-shot approach as a baseline using the full factorial method for 2 and 3 levels that yield a total of 33 and 244 sampling points. We compare both baselines with the accuracy of the ASAMS at a different number of sample points using MSE for an independent 35 point test set. Note that, with 75 samples, the algorithm performs better than the 244 one-shot sampling approach.

(58) $$\frac{1}{N}\sum_{N}^{i=1}{\left(Y^{i}-{\bar{Y}}^{\left(i,t\right)}\right)}^{2}).$$

**Figure 15:**
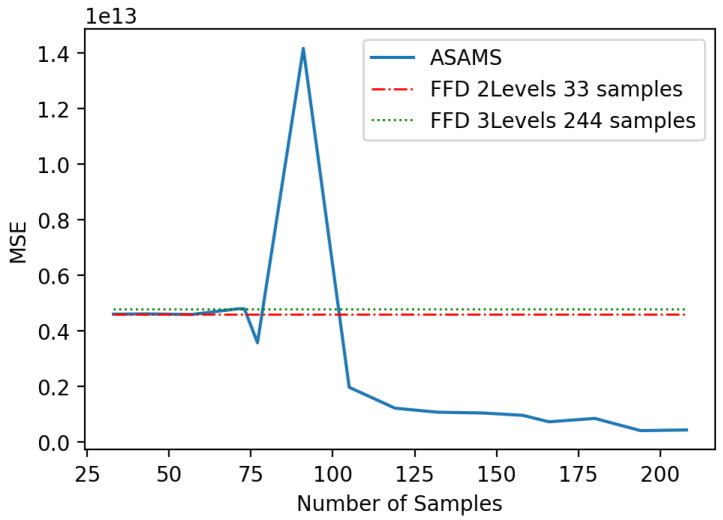
Interest region MSE comparison of the magnetic circuit.

As in the previous case, we can observe some oscillations in the ASAMS results. For this reason, we decided to perform also an independent testing set of 64 points of critical regions using ASAMS mechanisms. In Figure 15, a comparison of both baselines with the accuracy of the ASAMS using the Critical Testing set is shown. In this experiment, it can be seen that the 244 point baseline does not improve in the critical regions while the ASASM algorithm clearly improves without losing generalization.

**Figure 16:**
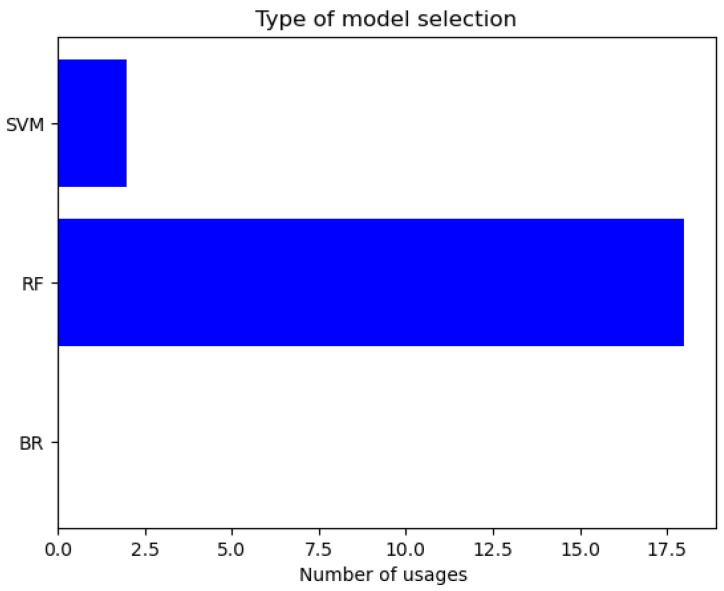
Type of model selection for the magnetic circuit.

A correct model selection and a fine hyperparameter tuning are crucial for a complex task. We hypothesize that the AutoML feature of the ASAMS will become increasingly important to improve the performance in harder problems. To analyze this hypothesis, we decide to register the number of changes between the models. In Figure 16, we show the changes between the model types.

**Figure 17:**
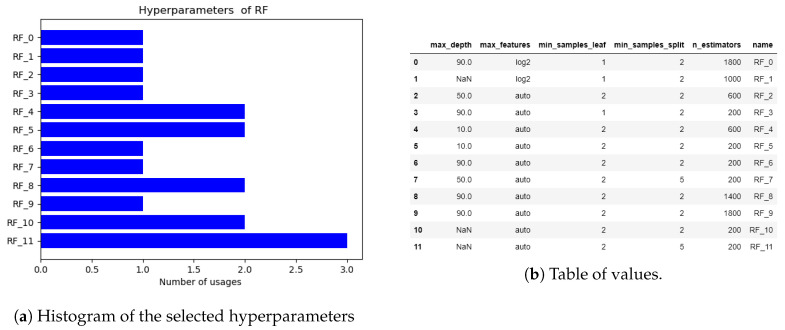
Histogram of the selected hyperparameters of the magnetic circuit.

Observe that in this case, the algorithm shows some changes between the model types. We are also interested in analyzing if the algorithm changes the hyperparameters for achieving a fine-tuning of the model. In Figure 17a, a histogram of the selected hyperparameters is shown. A table of the selected hyperparameters is shown in Figure 17b. In this analysis, we can appreciate that the algorithm adjusts the hyperparameters before and does not achieve convergence. The final model is RF with the following hyperparameters: max_depth = NaN, max_features = auto, min_samples_leaf = 2, min_samples_split = 5 and n_estimators = 200.

### 5.3. Discussion

We hypothesize that the capability of changing the algorithm of the surrogate model during the iterative adaptive sampling that generates additional data points will become crucial to improve the performance. While this was true in the magnetic circuit problem, in the nonlinear oscillator the model selection was not important after four iterations, and we can even argue that the changes between the selected models were minimum. From this behavior, we can infer that the additional computational cost is only justified in complex problems because in simpler problems the initial sampling provides enough information for selecting a suitable model for the task.

We decided to perform two different kinds of validation. The first one was performed using a random sampling of the valid input space. In this experiment, the baseline performs as expected when we use a bigger dataset the predictions of the model improved. As expected, the performance of the model trained using ASAMS was better than the baseline with fewer points in both cases. The second experiment was performed using a sampling of critical points form the model trained by ASAMS which by definition are the points in which our model performs worst. This dataset became interesting because we found out that the baseline models do not improve their performance when the size of the data set is increased as expected. However, the model train by ASAMS improves in the testing set when the data set size increases. This characteristic must be studied in future work.

After the analysis, we note that the use of ASMS should be limited to complex problems since the improvement is marginal in simple problems and does not justify the additional computational cost.

The proposed methodology can be generalized to any surrogation problems, provided there is some alternative for evaluating the performance of the proposed points. The evaluation can be performed online using any type of computational model or offline by an experimental method. In the case of experimental measures, the algorithm must be stopped in every iteration to preform experimental evaluations. From the target system, the designer must identify the inputs and outputs for the surrogate model. The inputs of the surrogate model will be selected as parameters, and they must be constrained. Then, the algorithm must start with a one-shot sampling method.

The ASAMS algorithm can be tuned by changing the number and type of candidate IA algorithms for surrogating the system, also the list of hyperparameters can be changed. Finally, the stop conditions, keep rate, max experiments, and the number of experiments must be set.

With all these hyperparameters the ASAMS algorithm can be tuned for many surrogation tasks.

## 6. Conclusions and Future Work

Adaptive sampling algorithms have been proven to be useful in reliability analysis for traditional surrogate models like the Kriging method, but the growing demand for algorithms to translate the surrogate models into applications such as digital twinning, design mining or soft sensors has generated the need to transfer adaptive sampling methods into machine learning models. In this paper, we have proposed a new adaptive sequential sampling approach that combines a meta-learning algorithm with two adaptive sampling methods for machine learning models. Constrain handling techniques were introduced to consider different types of discrete design parameters, allowing the algorithm to handle more types of problems. We proposed an elitism mechanism for reducing the number of candidate models as part of the meta-learning approach.

We selected two different study cases for testing the ASAMS performance: a benchmark and a real problem. In these tests, we compared the performance of a surrogate model trained with the ASAMS algorithm and a model trained with a baseline one-shot sampling. Two different testing sets were used for the comparison. The first one is a random sampling of 200 points and the other is a focal sampling of the critical points of the study case. These experiments show that the model generated by the ASAMS algorithm can obtain better performance than the baseline approaches with fewer sampled points. Additionally, we verified that the surrogate models were more robust to the critical parts of the target system without losing generalization.

Another important factor is the ability of the ASAMS methodology to perform a selection of suitable algorithms for surrogating the problem and the fine-tuning of the selected algorithm. In the test, we observed that this feature is relevant for complex models.

While the methodology was tested using a couple of well-known algorithms, it can be easily used for almost any machine learning algorithm and can be applied for generating a surrogate model for any field of study.

The ASAMS methodology is an initial approach to combine AutoML with adaptive sampling techniques. As future work, we suggest improving the proposed methodology with more complex AutoML algorithms. Additionally, we recommend studying further the differences between the critical points for a model and a system to improve the performance of adaptive sampling techniques.

## Figures and Tables

**Figure:**
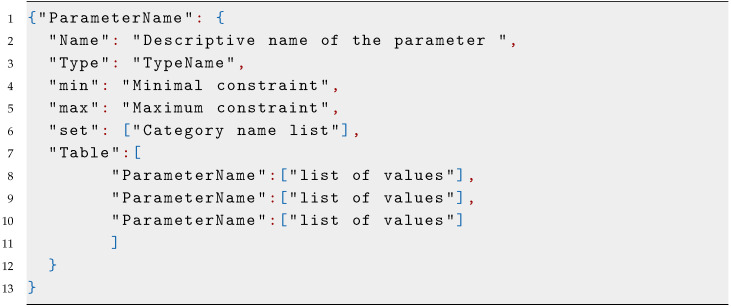
Listing 1: **Parameter JSON visualization.**

**Figure:**
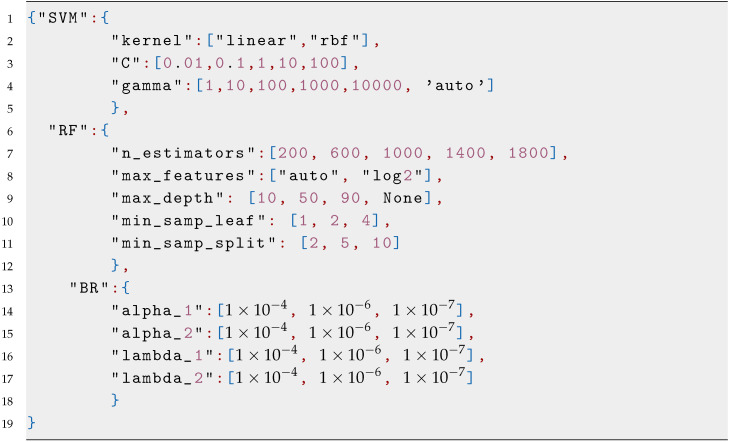
Listing 2: **Hyperparameter grid search.**

**Figure:**
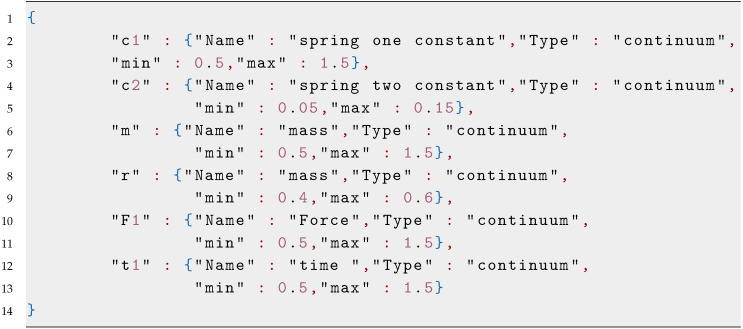
Listing 3: **Nonlinear oscillator parameters.**

**Figure:**
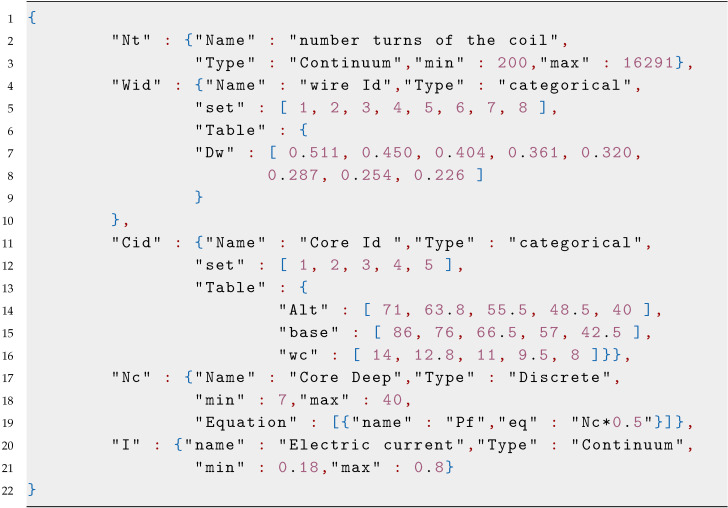
Listing 4: **Magneticcircuit parameters.**

## Nomenclatures

$Y^{i}$	Target vector of the *i*-th element of the Testing data set
$X_{i}$	Input vector of the *i*-th element of the Testing data set
${\bar{Y}}^{\left(i,t\right)}$	Output vector of the *t*-th surrogate model w/ the *i*-th input vector of the Testing data set
$F_{t}$	Algorithm selected from the $\hat{FT}$ algorithm set
$\hat{Hp}$	Set of all hyperparameter combinations for the *T* candidate solutions
${\hat{Hp}}_{t}$	Set of valid hyperparameter combinations for the *t*-th candidate model
$Hp_{\left(v,t\right)}$	Hyperparameters selected for the $F_{t}$ model
$hp_{\left(k_{t},t\right)}$	Value of the $k_{t}$ hyperparameter of the *t* model
$T_{d}$	Training dataset
$\hat{FT}$	Set of candidate algorithms
${\hat{P}}_{\left(k_{t},t\right)}$	Set of candidate values for the $k_{t}$ hyperparameter of the *t* model
$x_{\left(j,i\right)}$	*j*-th element of the input vector $X_{i}$
$y_{\left(s,i\right)}$	*s*-th element of the target vector $Y_{i}$
$d_{m}$	*m*-th data point of the Training dataset
$p_{\left(q_{\left(k,t\right)},k_{t},t\right)}$	*q*-th candidate value for the $k_{t}$ hyperparameter of the *t* model
$x_{j}^{L}$	Lower constraint for the *j*-th elements of the *i*-th input vector
$x_{j}^{U}$	Upper constraint for the *j*-th elements of the *i*-th input vector
*M*	Number of points of the training dataset
*N*	Number of points of the Testing dataset
*T*	Number of candidate algorithms
$K_{t}$	Number of hyperparameters of the *t*-th algorithm
*S*	Number of elements of the target vector
*J*	Number of elements of the input vector
$Q_{\left(k,t\right)}$	Number of candidate values of the $k_{t}$ hyperparameter of the *t* model
$V_{t}$	Number of valid hyperparameter combinations for the *t*-th model
